# Supplementary material for: Modeling glioblastoma heterogeneity as a dynamic network of cell states
Source: Mol Syst Biol. 2021 Sep 16;17(9):e10105. doi: 10.15252/msb.202010105 (PMC8444284; doi:10.15252/msb.202010105)
Supplement: Supplementary file 6 — Source Data for Figure 5 [file MSB-17-e10105-s004.zip › Figure5A_sourcedata/GSEA_3071/nosigdb_stateII.GseaPreranked.1622795151171/FELDMAN_2020_G2-M.html]

Details for gene set FELDMAN\_2020\_G2-M[GSEA]

|  || Dataset | state23071 |
| Phenotype | NoPhenotypeAvailable |
| Upregulated in class | na\_pos |
| GeneSet | FELDMAN\_2020\_G2-M |
| Enrichment Score (ES) | 0.48437437 |
| Normalized Enrichment Score (NES) | 3.3189063 |
| Nominal p-value | 0.0 |
| FDR q-value | 0.0 |
| FWER p-Value | 0.0 |
Table: GSEA Results Summary

  

Fig 1: Enrichment plot: FELDMAN\_2020\_G2-M      
 Profile of the Running ES Score & Positions of GeneSet Members on the Rank Ordered List

  

| PROBE | GENE SYMBOL | GENE\_TITLE | RANK IN GENE LIST | RANK METRIC SCORE | RUNNING ES | CORE ENRICHMENT || 1 | RRM2 |  |  | 3 | 1.857 | 0.0102 | Yes |
| 2 | ANLN |  |  | 4 | 1.760 | 0.0206 | Yes |
| 3 | CDK1 |  |  | 14 | 1.540 | 0.0274 | Yes |
| 4 | DIAPH3 |  |  | 15 | 1.531 | 0.0365 | Yes |
| 5 | ZWINT |  |  | 16 | 1.510 | 0.0454 | Yes |
| 6 | HJURP |  |  | 17 | 1.490 | 0.0542 | Yes |
| 7 | ANKRD1 |  |  | 21 | 1.446 | 0.0620 | Yes |
| 8 | UBE2C |  |  | 23 | 1.438 | 0.0703 | Yes |
| 9 | CENPK |  |  | 26 | 1.382 | 0.0779 | Yes |
| 10 | FBXO5 |  |  | 30 | 1.359 | 0.0852 | Yes |
| 11 | TK1 |  |  | 42 | 1.281 | 0.0900 | Yes |
| 12 | AURKB |  |  | 44 | 1.276 | 0.0973 | Yes |
| 13 | CCNA2 |  |  | 53 | 1.230 | 0.1025 | Yes |
| 14 | SKA3 |  |  | 57 | 1.215 | 0.1089 | Yes |
| 15 | MAD2L1 |  |  | 61 | 1.199 | 0.1152 | Yes |
| 16 | UBE2T |  |  | 62 | 1.192 | 0.1223 | Yes |
| 17 | SPC25 |  |  | 68 | 1.156 | 0.1278 | Yes |
| 18 | MELK |  |  | 69 | 1.145 | 0.1346 | Yes |
| 19 | TOP2A |  |  | 72 | 1.132 | 0.1408 | Yes |
| 20 | KIF2C |  |  | 73 | 1.132 | 0.1475 | Yes |
| 21 | FOSL1 |  |  | 77 | 1.119 | 0.1534 | Yes |
| 22 | GMNN |  |  | 83 | 1.108 | 0.1586 | Yes |
| 23 | CEP55 |  |  | 87 | 1.096 | 0.1643 | Yes |
| 24 | KIFC1 |  |  | 88 | 1.094 | 0.1708 | Yes |
| 25 | NDC80 |  |  | 94 | 1.074 | 0.1759 | Yes |
| 26 | LRR1 |  |  | 98 | 1.060 | 0.1814 | Yes |
| 27 | TMPO |  |  | 99 | 1.056 | 0.1876 | Yes |
| 28 | NCAPG |  |  | 100 | 1.056 | 0.1939 | Yes |
| 29 | MKI67 |  |  | 107 | 1.031 | 0.1984 | Yes |
| 30 | TPX2 |  |  | 111 | 1.025 | 0.2037 | Yes |
| 31 | CDCA8 |  |  | 113 | 1.018 | 0.2095 | Yes |
| 32 | TRIP13 |  |  | 114 | 1.014 | 0.2155 | Yes |
| 33 | KIF11 |  |  | 115 | 1.014 | 0.2215 | Yes |
| 34 | SMC2 |  |  | 123 | 1.005 | 0.2257 | Yes |
| 35 | C12orf75 |  |  | 124 | 1.004 | 0.2316 | Yes |
| 36 | NUF2 |  |  | 125 | 1.004 | 0.2375 | Yes |
| 37 | DEPDC1 |  |  | 126 | 0.999 | 0.2435 | Yes |
| 38 | CKS1B |  |  | 128 | 0.999 | 0.2491 | Yes |
| 39 | TACC3 |  |  | 130 | 0.992 | 0.2547 | Yes |
| 40 | KIF20B |  |  | 133 | 0.989 | 0.2601 | Yes |
| 41 | ESCO2 |  |  | 144 | 0.971 | 0.2632 | Yes |
| 42 | CKAP2L |  |  | 148 | 0.959 | 0.2681 | Yes |
| 43 | RANBP1 |  |  | 149 | 0.958 | 0.2738 | Yes |
| 44 | KIF23 |  |  | 161 | 0.936 | 0.2765 | Yes |
| 45 | SHCBP1 |  |  | 164 | 0.933 | 0.2815 | Yes |
| 46 | RTKN2 |  |  | 166 | 0.931 | 0.2868 | Yes |
| 47 | GTSE1 |  |  | 167 | 0.929 | 0.2923 | Yes |
| 48 | CDCA2 |  |  | 171 | 0.922 | 0.2970 | Yes |
| 49 | CENPM |  |  | 178 | 0.901 | 0.3008 | Yes |
| 50 | FAM83D |  |  | 197 | 0.876 | 0.3013 | Yes |
| 51 | SPDL1 |  |  | 199 | 0.874 | 0.3062 | Yes |
| 52 | H2AFZ |  |  | 213 | 0.864 | 0.3080 | Yes |
| 53 | PBK |  |  | 214 | 0.864 | 0.3131 | Yes |
| 54 | CDCA3 |  |  | 219 | 0.858 | 0.3172 | Yes |
| 55 | CENPH |  |  | 221 | 0.855 | 0.3220 | Yes |
| 56 | BUB1B |  |  | 223 | 0.851 | 0.3268 | Yes |
| 57 | ASPM |  |  | 231 | 0.842 | 0.3299 | Yes |
| 58 | HMGA1 |  |  | 238 | 0.834 | 0.3333 | Yes |
| 59 | MIS18A |  |  | 239 | 0.833 | 0.3383 | Yes |
| 60 | DBF4 |  |  | 240 | 0.832 | 0.3432 | Yes |
| 61 | RACGAP1 |  |  | 241 | 0.831 | 0.3481 | Yes |
| 62 | PRC1 |  |  | 242 | 0.830 | 0.3530 | Yes |
| 63 | TUBB6 |  |  | 244 | 0.828 | 0.3577 | Yes |
| 64 | LMNB1 |  |  | 251 | 0.824 | 0.3610 | Yes |
| 65 | TUBB4B |  |  | 271 | 0.784 | 0.3608 | Yes |
| 66 | HMGB2 |  |  | 276 | 0.780 | 0.3643 | Yes |
| 67 | SMC4 |  |  | 279 | 0.777 | 0.3684 | Yes |
| 68 | CENPE |  |  | 284 | 0.775 | 0.3720 | Yes |
| 69 | GLIPR1 |  |  | 296 | 0.758 | 0.3736 | Yes |
| 70 | SERPINE1 |  |  | 299 | 0.756 | 0.3776 | Yes |
| 71 | DEK |  |  | 302 | 0.754 | 0.3815 | Yes |
| 72 | CENPA |  |  | 303 | 0.751 | 0.3860 | Yes |
| 73 | KIF4A |  |  | 309 | 0.746 | 0.3891 | Yes |
| 74 | CKS2 |  |  | 312 | 0.745 | 0.3930 | Yes |
| 75 | PRR11 |  |  | 314 | 0.743 | 0.3972 | Yes |
| 76 | CENPN |  |  | 320 | 0.737 | 0.4002 | Yes |
| 77 | ANP32B |  |  | 325 | 0.734 | 0.4035 | Yes |
| 78 | CENPW |  |  | 330 | 0.727 | 0.4068 | Yes |
| 79 | SPC24 |  |  | 340 | 0.719 | 0.4088 | Yes |
| 80 | ARHGAP11A |  |  | 346 | 0.712 | 0.4117 | Yes |
| 81 | AURKA |  |  | 360 | 0.705 | 0.4125 | Yes |
| 82 | DTYMK |  |  | 364 | 0.701 | 0.4159 | Yes |
| 83 | TTK |  |  | 365 | 0.701 | 0.4200 | Yes |
| 84 | EIF5 |  |  | 366 | 0.701 | 0.4242 | Yes |
| 85 | TMEM106C |  |  | 377 | 0.694 | 0.4257 | Yes |
| 86 | KIF14 |  |  | 378 | 0.693 | 0.4298 | Yes |
| 87 | PARPBP |  |  | 382 | 0.688 | 0.4331 | Yes |
| 88 | KIF18A |  |  | 392 | 0.683 | 0.4349 | Yes |
| 89 | MT2A |  |  | 393 | 0.682 | 0.4389 | Yes |
| 90 | CCDC34 |  |  | 411 | 0.672 | 0.4385 | Yes |
| 91 | SAE1 |  |  | 418 | 0.669 | 0.4409 | Yes |
| 92 | KPNA2 |  |  | 420 | 0.669 | 0.4446 | Yes |
| 93 | FOXM1 |  |  | 423 | 0.667 | 0.4481 | Yes |
| 94 | RAN |  |  | 424 | 0.667 | 0.4520 | Yes |
| 95 | BUB3 |  |  | 441 | 0.657 | 0.4518 | Yes |
| 96 | PIF1 |  |  | 445 | 0.653 | 0.4549 | Yes |
| 97 | NCAPD2 |  |  | 457 | 0.647 | 0.4559 | Yes |
| 98 | SNRPB |  |  | 467 | 0.642 | 0.4574 | Yes |
| 99 | HMMR |  |  | 475 | 0.639 | 0.4593 | Yes |
| 100 | RHEB |  |  | 500 | 0.629 | 0.4569 | Yes |
| 101 | BIRC5 |  |  | 505 | 0.628 | 0.4596 | Yes |
| 102 | ECT2 |  |  | 510 | 0.626 | 0.4623 | Yes |
| 103 | SNRPD1 |  |  | 523 | 0.615 | 0.4628 | Yes |
| 104 | RPA3 |  |  | 532 | 0.611 | 0.4644 | Yes |
| 105 | DDX39A |  |  | 542 | 0.605 | 0.4657 | Yes |
| 106 | MEST |  |  | 563 | 0.593 | 0.4640 | Yes |
| 107 | MIS18BP1 |  |  | 574 | 0.588 | 0.4649 | Yes |
| 108 | CENPF |  |  | 575 | 0.588 | 0.4684 | Yes |
| 109 | CCNB1 |  |  | 579 | 0.587 | 0.4711 | Yes |
| 110 | PDLIM7 |  |  | 580 | 0.587 | 0.4746 | Yes |
| 111 | EXOSC8 |  |  | 604 | 0.576 | 0.4721 | Yes |
| 112 | CACYBP |  |  | 613 | 0.573 | 0.4734 | Yes |
| 113 | TUBG1 |  |  | 617 | 0.570 | 0.4760 | Yes |
| 114 | CKAP2 |  |  | 619 | 0.569 | 0.4791 | Yes |
| 115 | SPAG5 |  |  | 642 | 0.555 | 0.4768 | Yes |
| 116 | NUSAP1 |  |  | 654 | 0.550 | 0.4772 | Yes |
| 117 | CCT5 |  |  | 662 | 0.548 | 0.4786 | Yes |
| 118 | H2AFX |  |  | 671 | 0.544 | 0.4798 | Yes |
| 119 | TUBB |  |  | 678 | 0.538 | 0.4815 | Yes |
| 120 | SLC25A5 |  |  | 680 | 0.537 | 0.4844 | Yes |
| 121 | DLGAP5 |  |  | 712 | 0.529 | 0.4795 | No |
| 122 | GPSM2 |  |  | 719 | 0.526 | 0.4811 | No |
| 123 | CCDC18 |  |  | 735 | 0.520 | 0.4803 | No |
| 124 | PHF19 |  |  | 745 | 0.518 | 0.4811 | No |
| 125 | PTGES3 |  |  | 763 | 0.512 | 0.4797 | No |
| 126 | CMC2 |  |  | 785 | 0.503 | 0.4773 | No |
| 127 | CDC20 |  |  | 844 | 0.486 | 0.4653 | No |
| 128 | TUBA1B |  |  | 862 | 0.481 | 0.4638 | No |
| 129 | DEPDC1B |  |  | 906 | 0.470 | 0.4555 | No |
| 130 | KIF22 |  |  | 975 | 0.450 | 0.4407 | No |
| 131 | HSP90AA1 |  |  | 1008 | 0.443 | 0.4351 | No |
| 132 | PLK1 |  |  | 1009 | 0.442 | 0.4377 | No |
| 133 | NUP35 |  |  | 1020 | 0.438 | 0.4377 | No |
| 134 | POC1A |  |  | 1080 | 0.421 | 0.4251 | No |
| 135 | CDKN1A |  |  | 1088 | 0.419 | 0.4257 | No |
| 136 | NEK2 |  |  | 1100 | 0.417 | 0.4254 | No |
| 137 | CYCS |  |  | 1113 | 0.415 | 0.4248 | No |
| 138 | TCP1 |  |  | 1116 | 0.414 | 0.4267 | No |
| 139 | BUB1 |  |  | 1145 | 0.408 | 0.4219 | No |
| 140 | MZT1 |  |  | 1219 | 0.393 | 0.4055 | No |
| 141 | STIP1 |  |  | 1227 | 0.392 | 0.4060 | No |
| 142 | PTMA |  |  | 1233 | 0.390 | 0.4070 | No |
| 143 | PSMC3 |  |  | 1241 | 0.389 | 0.4075 | No |
| 144 | STMN1 |  |  | 1249 | 0.387 | 0.4080 | No |
| 145 | BCL2L12 |  |  | 1252 | 0.386 | 0.4098 | No |
| 146 | ANP32E |  |  | 1334 | 0.371 | 0.3912 | No |
| 147 | RNASEH2A |  |  | 1341 | 0.369 | 0.3918 | No |
| 148 | LDHA |  |  | 1374 | 0.361 | 0.3857 | No |
| 149 | KNSTRN |  |  | 1458 | 0.346 | 0.3664 | No |
| 150 | CLIC1 |  |  | 1552 | 0.331 | 0.3445 | No |
| 151 | CDC27 |  |  | 1562 | 0.330 | 0.3441 | No |
| 152 | TIMM10 |  |  | 1567 | 0.329 | 0.3451 | No |
| 153 | TMEM237 |  |  | 1576 | 0.328 | 0.3449 | No |
| 154 | CALM2 |  |  | 1625 | 0.320 | 0.3345 | No |
| 155 | LSM4 |  |  | 1676 | 0.313 | 0.3235 | No |
| 156 | HMGB1 |  |  | 1720 | 0.308 | 0.3143 | No |
| 157 | RUVBL2 |  |  | 1779 | 0.301 | 0.3011 | No |
| 158 | CTGF |  |  | 1782 | 0.301 | 0.3024 | No |
| 159 | REEP4 |  |  | 1786 | 0.300 | 0.3034 | No |
| 160 | OIP5 |  |  | 1789 | 0.299 | 0.3047 | No |
| 161 | ILF2 |  |  | 1833 | 0.294 | 0.2954 | No |
| 162 | SRSF3 |  |  | 1834 | 0.294 | 0.2971 | No |
| 163 | RBM8A |  |  | 1971 | 0.276 | 0.2638 | No |
| 164 | MPHOSPH9 |  |  | 2082 | 0.264 | 0.2371 | No |
| 165 | CKAP5 |  |  | 2135 | 0.258 | 0.2252 | No |
| 166 | MXD3 |  |  | 2156 | 0.256 | 0.2216 | No |
| 167 | CBX1 |  |  | 2210 | 0.251 | 0.2095 | No |
| 168 | RAB13 |  |  | 2381 | -0.268 | 0.1674 | No |
| 169 | SHISA4 |  |  | 2493 | -0.284 | 0.1405 | No |
| 170 | ATP6V0E1 |  |  | 2545 | -0.293 | 0.1292 | No |
| 171 | FTH1 |  |  | 2672 | -0.312 | 0.0986 | No |
| 172 | CCDC167 |  |  | 2753 | -0.327 | 0.0800 | No |
| 173 | GAS2L3 |  |  | 2755 | -0.327 | 0.0817 | No |
| 174 | IGFBP5 |  |  | 2820 | -0.339 | 0.0672 | No |
| 175 | MIAT |  |  | 2996 | -0.371 | 0.0245 | No |
| 176 | NUDCD2 |  |  | 3003 | -0.373 | 0.0251 | No |
| 177 | COMMD6 |  |  | 3174 | -0.421 | -0.0161 | No |
| 178 | PDLIM4 |  |  | 3220 | -0.433 | -0.0251 | No |
| 179 | PLD3 |  |  | 3285 | -0.452 | -0.0389 | No |
| 180 | EIF4A2 |  |  | 3289 | -0.453 | -0.0369 | No |
| 181 | TCEAL8 |  |  | 3376 | -0.488 | -0.0562 | No |
| 182 | METRN |  |  | 3468 | -0.526 | -0.0764 | No |
| 183 | ARL6IP1 |  |  | 3574 | -0.586 | -0.0999 | No |
| 184 | IFITM3 |  |  | 3578 | -0.589 | -0.0972 | No |
| 185 | SAT1 |  |  | 3659 | -0.653 | -0.1139 | No |
| 186 | PRCP |  |  | 3775 | -0.766 | -0.1390 | No |
| 187 | BEX4 |  |  | 3796 | -0.785 | -0.1394 | No |
| 188 | SOX4 |  |  | 3797 | -0.786 | -0.1348 | No |
| 189 | TTYH1 |  |  | 3807 | -0.795 | -0.1324 | No |
| 190 | RBP1 |  |  | 3878 | -0.898 | -0.1451 | No |
| 191 | PSAP |  |  | 3882 | -0.907 | -0.1405 | No |
| 192 | TM7SF2 |  |  | 3918 | -0.999 | -0.1436 | No |
| 193 | HES6 |  |  | 3961 | -1.092 | -0.1479 | No |
| 194 | PCSK1N |  |  | 3987 | -1.179 | -0.1473 | No |
| 195 | ITM2B |  |  | 3995 | -1.225 | -0.1419 | No |
| 196 | FGFBP3 |  |  | 3999 | -1.237 | -0.1353 | No |
| 197 | NOVA1 |  |  | 4008 | -1.312 | -0.1296 | No |
| 198 | TNC |  |  | 4039 | -1.549 | -0.1282 | No |
| 199 | LIMA1 |  |  | 4056 | -1.716 | -0.1221 | No |
| 200 | IGFBP3 |  |  | 4058 | -1.726 | -0.1122 | No |
| 201 | CLU |  |  | 4069 | -1.908 | -0.1035 | No |
| 202 | SCRG1 |  |  | 4070 | -2.021 | -0.0915 | No |
| 203 | BEX1 |  |  | 4072 | -2.034 | -0.0797 | No |
| 204 | PTN |  |  | 4077 | -2.187 | -0.0678 | No |
| 205 | S100B |  |  | 4081 | -2.383 | -0.0545 | No |
| 206 | GPM6B |  |  | 4083 | -2.435 | -0.0403 | No |
| 207 | FABP7 |  |  | 4095 | -3.351 | -0.0233 | No |
| 208 | PTPRZ1 |  |  | 4097 | -4.027 | 0.0003 | No |
Table: GSEA details [plain text format]

  

Fig 2: FELDMAN\_2020\_G2-M: Random ES distribution      
 Gene set null distribution of ES for **FELDMAN\_2020\_G2-M**
